# A Novel Strategy to Enhance Microfracture Treatment With Stromal Cell-Derived Factor-1 in a Rat Model

**DOI:** 10.3389/fcell.2020.595932

**Published:** 2021-02-04

**Authors:** Taylor Mustapich, John Schwartz, Pablo Palacios, Haixiang Liang, Nicholas Sgaglione, Daniel A. Grande

**Affiliations:** ^1^Orthopaedic Research Laboratory, Feinstein Institutes for Medical Research, Northwell Health, Manhasset, NY, United States; ^2^Department of Orthopaedic Surgery, Northwell Health, New Hyde Park, NY, United States

**Keywords:** osteoarthritis-therapies < cartilage, subchondral arthroplasty, tissue scaffolds, mesenchymal stem cell, microfracture (MFX), stromal cell derived factor-1 (CXCL12), bone marrow stem cell-based therapy

## Abstract

**Background:**

Microfracture is one of the most widely used techniques for the repair of articular cartilage. However, microfracture often results in filling of the chondral defect with fibrocartilage, which exhibits poor durability and sub-optimal mechanical properties. Stromal cell-derived factor-1 (SDF-1) is a potent chemoattractant for mesenchymal stem cells (MSCs) and is expressed at high levels in bone marrow adjacent to developing cartilage during endochondral bone formation. Integrating SDF-1 into an implantable collagen scaffold may provide a chondro-conductive and chondro-inductive milieu *via* chemotaxis of MSCs and promotion of chondrogenic differentiation, facilitating more robust hyaline cartilage formation following microfracture.

**Objective:**

This work aimed to confirm the chemoattractive properties of SDF-1 *in vitro* and develop a one-step method for incorporating SDF-1 *in vivo* to enhance cartilage repair using a rat osteochondral defect model.

**Methods:**

Bone marrow-derived MSCs (BMSCs) were harvested from the femurs of Sprague–Dawley rats and cultured in low-glucose Dulbecco’s modified Eagle’s medium containing 10% fetal bovine serum, with the medium changed every 3 days. Passage 1 MSCs were analyzed by flow cytometry with an S3 Cell Sorter (Bio-Rad). *In vitro* cell migration assays were performed on MSCs by labeling cells with carboxyfluorescein diacetate, succinimidyl ester (CFDA-SE; Bio-Rad). For the microfracture model, a 1.6-mm-diameter osteochondral defect was created in the femoral trochleae of 20 Sprague–Dawley rats bilaterally until bone marrow spillage was seen under saline irrigation. One knee was chosen at random to receive implantation of the scaffold, and the contralateral knee was left unfilled as an empty control. Type I collagen scaffolds (Kensey Nash) were coated with either gelatin only or gelatin and SDF-1 using a dip coating process. The rats received implantation of either a gelatin-only scaffold (*N* = 10) or gelatin-and-SDF-1 scaffold (*N* = 10) at the site of the microfracture. Femurs were collected for histological analyses at 4- and 8-week time points post-operatively, and sections were stained with Safranin O/Fast Green. The samples were graded blindly by two observers using the Modified O’Driscoll score, a validated scoring system for chondral repair. A minimum of 10 separate grading scores were made per sample and averaged. Quantitative comparisons of cell migration *in vitro* were performed with one-way ANOVA. Cartilage repair *in vivo* was also compared among groups with one-way ANOVA, and the results were presented as mean ± standard deviation, with *P*-values < 0.05 considered as statistically significant.

**Results:**

MSC migration showed a dose–response relationship with SDF-1, with an optimal dosage for chemotaxis between 10 and 100 ng/ml. After scaffold implantation, the SDF-1-treated group demonstrated complete filling of the cartilage defect with mature cartilage tissue, exhibiting strong proteoglycan content, smooth borders, and good incorporation into marginal cartilage. Modified O’Driscoll scores after 8 weeks showed a significant improvement of cartilage repair in the SDF-1 group relative to the empty control group (*P* < 0.01), with a trend toward improvement when compared with the gelatin-only-scaffold group (*P* < 0.1). No significant differences in scores were found between the empty defect group and gelatin-only group.

**Conclusion:**

In this study, we demonstrated a simple method for improving the quality of cartilage defect repair in a rat model of microfracture. We confirmed the chemotactic properties of SDF-1 on rat MSCs and found an optimized dosage range for chemotaxis between 10 and 100 ng/ml. Furthermore, we demonstrated a strategy to incorporate SDF-1 into gelatin–collagen I scaffolds *in vivo* at the site of an osteochondral defect. SDF-1-treated defects displayed robust hyaline cartilage resurfacing of the defect with minimal fibrous tissue, in contrast to the empty control group. The results of the *in vitro* and *in vivo* studies together suggest that SDF-1-mediated signaling may significantly improve the quality of cartilage regeneration in an osteochondral defect.

## Introduction

While the etiology of joint degeneration and osteoarthritis occurs at the molecular, cellular, and tissue level, current treatments of isolated chondral defects are primarily surgical (Furman et al., 2006; Daher et al., 2009; Anderson et al., 2011). Regeneration of hyaline cartilage is limited by the lack of chondral vascularity, diminishing the recruitment of renewable cells to the site of injury (Csaki et al., 2008; Fong et al., 2011). Thus, penetration of the subchondral bone with techniques such as microfracture emerged and served to recruit mesenchymal stem cells (MSCs) from the bone marrow to infiltrate the overlying chondral lesion. However, long-term durability was inadequate, as the native hyaline cartilage was repaired with fibrocartilage consisting of primarily type I collagen, which is prone to rapid degeneration and less adaptable than its hyaline counterpart (Mithoefer et al., 2009; Gomoll and Minas, 2014; Richter et al., 2016). It is thought that microfracture alone does not recruit a sufficient amount of reparative cells and growth factors to promote adequate native tissue repair (Richter, 2009). One potential therapeutic strategy to augment the chondrogenic milieu following microfracture is by incorporating homing factors into cell-free scaffolds to promote the recruitment of MSCs to the site of injury (Mishima and Lotz, 2008; Chanda et al., 2010; Fong et al., 2011; Eseonu and De Bari, 2015; Truong et al., 2017; Lu et al., 2018).

Stromal cell-derived factor-1 (SDF-1) is a potent chemoattractant for MSCs and has associations with bone repair (Hwang et al., 2015; Liu et al., 2015). SDF-1 attracts MSCs to the site of bone fractures and is expressed in marrow adjacent to developing cartilage during endochondral bone formation (Wei et al., 2010; Murata et al., 2012; Toupadakis et al., 2012). In addition, the receptor for SDF-1, C-X-C chemokine receptor type 4 (CXCR4), plays a significant role in chondrocyte differentiation and proliferation (Mazzetti et al., 2004). Integrating SDF-1 into an implantable collagen scaffold may improve the quality of cartilage repair by recruiting MSCs from the underlying bone marrow and promoting a chondroinductive milieu. We seek to evaluate a novel method of improving the quality of chondral repair following microfracture by incorporating SDF-1 into an implantable, cell-free scaffold and directly comparing it to microfracture alone.

## Materials and Methods

### Preparation of BMSCs

Rat bone marrow-derived MSCs (BMSCs) were isolated from the femurs of Sprague–Dawley rats as previously described (Haynesworth et al., 1992). Briefly, the femurs were harvested using an aseptic technique. After removing the soft tissue and periosteum, the bones were cut using a bone rongeur. Bone marrow was washed out with low-glucose Dulbecco’s modified Eagle’s medium (Fisher Scientific, Pittsburgh, PA, United States) containing 10% fetal bovine serum (Fisher Scientific, Pittsburgh, PA, United States) using a syringe. The bone marrow was then mixed with medium and cultured at 37°C with 5% CO_2_ in an incubator, with the medium changed every 3 days.

### Flow Cytometry Study

Rat BMSCs at passage 1 were detached from the cell culture flask by Accutase (Innovative Cell Technologies, San Diego, CA, United States). After washing with phosphate-buffered saline (PBS), the cells were stained with antibodies against CD45 (AbD Serotec, Raleigh, NC, United States), CD73 (BD Biosciences, San Jose, CA, United States), CD90 (BioLegend, San Diego, CA, United States), and CD106 (BioLegend, San Diego, CA, United States). The stained cells were analyzed by flow cytometry with an S3 Cell Sorter (Bio-Rad, Hercules, CA, United States).

### *In vitro* Cell Migration Assay

Rat BMSCs at passage 1 were serum-starved for 2 h at 37°C. A 24-well plate was loaded with Transwell inserts (8.0 μm in pore size; Fisher Scientific, Pittsburgh, PA, United States). The lower chambers contained 600 μl of either serum-free medium (negative control), medium with serum (positive control), or SDF-1 (1, 10, or 100 ng/ml; R&D Systems, Minneapolis, MN, United States). Each condition was set up in triplicate. The serum-starved BMSCs (105 cells/well) were loaded on the upper chamber and incubated at 37°C for 4 h. The membranes were fixed with formalin for 10 min and washed with PBS before staining with Wright–Giemsa. The migrated cells were counted under a light microscope (×400), with 30 random fields analyzed per membrane. The results are represented as the average number of migrated cells counted per ×400 magnification field.

For morphological observation, BMSCs were labeled with carboxyfluorescein diacetate, succinimidyl ester (CFDA-SE; Bio-Rad, Hercules, CA, United States) following the manufacturer’s instructions prior to loading on Transwell inserts. Fluorescence blocking inserts were used; thus, fluorescence was visible only when cells migrated to the observation side of the membrane.

### Scaffold Preparation

Type I collagen was selected as the scaffold biomaterial given its proven safety and biocompatibility profile and widespread availability, making it an ideal agent for translation into clinical application (Tampieri et al., 2008; Kon et al., 2010). Collagen I scaffolds (Kensey Nash, Exton, PA, United States) were coated with either gelatin only (control) or gelatin and SDF-1 using a dip coating process previously described (Dines et al., 2007, 2011; Uggen et al., 2010; Cummings et al., 2012). Briefly, gelatin was prepared by heating a 10% (wt) solution of medical-grade soluble bovine collagen (Semed-S, Kensey-Nash, Exton PA) to 80°C for 10 min, followed by incubation at 37°C. SDF-1 (50 ng/ml) was reconstituted in 10 mM HCl and mixed with the gelatin at a ratio of 2:1 (gelatin/SDF). Collagen scaffolds were pre-treated in 70% EtOH for 10 min, washed with PBS, and then dip-coated in the gelatin solution at 37°C for 30 min with gentle agitation. The scaffolds were removed from the solution and air-dried overnight in a sterile laminar flow hood.

### Surgical Procedure

The usage of animals in this study was approved by the Institutional Animal Care and Use Committee of Feinstein Institutes for Medical Research. A medial parapatellar approach to the knee joint was performed bilaterally in Sprague–Dawley rats (*N* = 20; weight, 500 g). A 1.6-mm-diameter full-thickness osteochondral defect was created in each femoral trochlea until bone marrow spillage was visualized under saline irrigation as a model of microfracture. One knee was chosen at random with a coin toss to receive scaffold implantation (1.8 mm in diameter, 1.5 mm in thickness) and the contralateral knee served as an empty control. The rats received implantation of a scaffold dip-coated either with gelatin only (*N* = 10) or with gelatin and SDF-1 (*N* = 10). Femurs were collected for histological analysis at 4- and 8-week time points post-operatively, with the endpoints defined based upon current literature (Lee and Im, 2012; Dahlin et al., 2014; Mahmoud et al., 2017; Hayashi et al., 2018).

### Histological Evaluation

The distal femurs were dissected free of soft tissue and fixed in 10% Millonig’s buffered formalin (Fisher Scientific, Pittsburgh, PA, United States). The samples were decalcified with formic acid decalcification solution (Fisher Scientific, Pittsburgh, PA, United States) for 5 days with shaking, then embedded in paraffin, and cut in the coronal plane (5 μm in thickness). Serial sections were cut at 100-μm intervals through an area approximately 30% of the total surface area of the defect. The sections were mounted and stained with Safranin-O/Fast Green. The samples were graded blindly by two observers using the Modified O’Driscoll Score, a validated scoring system for chondral repair (Qi et al., 2014). A minimum of 10 separate grading scores were made per sample and averaged.

### Statistical Analyses

The samples were tested for normality using the Shapiro–Wilk test. Quantitative comparisons of cell migration *in vitro* were performed with one-way ANOVA. The results are presented as mean ± standard deviation, with *P*-values less than 0.05 considered as statistically significant. Cartilage repair *in vivo* was compared among groups with one-way ANOVA. The results are presented as mean ± standard deviation, with *P*-values less than 0.05 considered as statistically significant.

## Results

### Flow Cytometric Characteristics of Rat BMSCs

**FIGURE 1:**
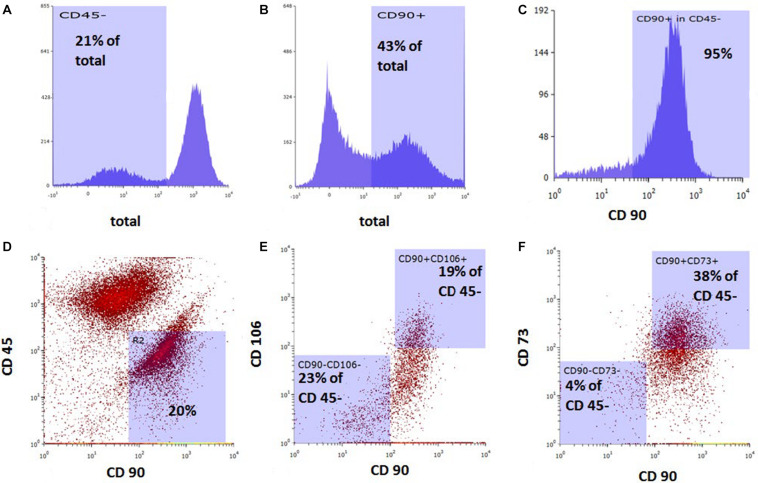
Flow cytometric analysis of rat bone marrow-derived mesenchymal stem cells prior to *in vitro* cell migration studies. Cell population stained with **(A)** anti-CD45, **(B)** anti-CD90, and **(C)** anti-CD90 among CD45-negative cells. Dot plot of combined staining for **(D)** CD45 and CD90, **(E)** CD106 and CD90 among CD45-negative population, and **(F)** CD73 and CD90 among CD45-negative population.

Bone marrow-derived MSCs at passage 1 were 43% CD90-positive and 21% CD45-negative. Among the CD45-negative cells, 95% were CD90-positive, 19% of CD45-negative cells were positive for both CD90 and CD106, and 38% of CD45-negative cells were positive for both CD90 and CD73 (Figure 1).

### *In vitro* Cell Migration

**FIGURE 2:**
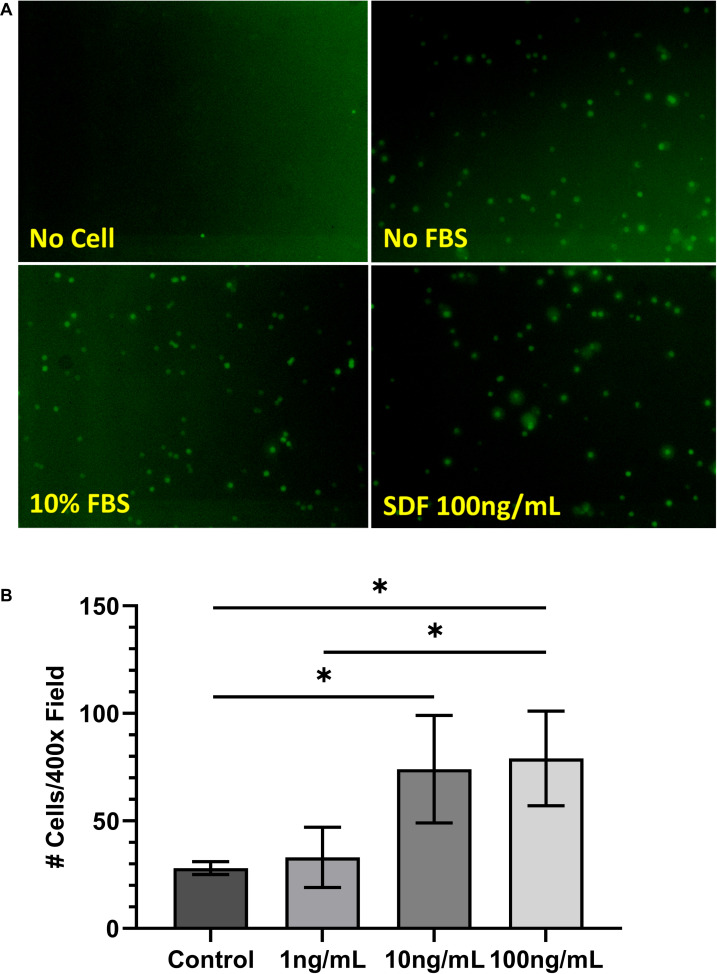
**(A)** Morphology of carboxyfluorescein diacetate–succinimidyl ester-stained bone marrow-derived mesenchymal stem cells (BMSCs) following cell migration assay. **(B)** Results of cell migration assay of BMSCs treated with stromal cell-derived factor-1 (1, 10, and 100 ng/ml). Values are represented as average number of cells counted per ×400 field. ^∗^*P* < 0.05.

Chemoattraction of BMSCs by SDF-1 was tested with the Transwell culture system *in vitro*. After 4 h in culture, the CFDA-SE-stained cells were found to have migrated in great quantities toward the SDF-1-containing chamber (100 ng/ml). Morphologically, these cells were round and larger than the pore size (Figure 2A).

To determine the optimal dosage of SDF-1 for chemoattraction of BMSCs, SDF-1 was added to the Transwell system at concentrations of 1, 10, and 100 ng/ml. Cell migration was increased significantly from the control group at both 10 ng/ml (28 ± 3 *vs*. 74 ± 25 cells/×400 field, respectively; *P* = 0.04) and 100 ng/ml (79 ± 22 cells/×400 field; *P* = 0.04) concentrations of SDF-1, which is consistent with previous literature (Figure 2B) (Ji et al., 2013). Cell migration did not differ significantly between the control group and the SDF-1 group containing 1 ng/ml (33 ± 14 cells/×400 field; *P* = 0.56).

### *In vivo* Cartilage Repair

**FIGURE 3:**
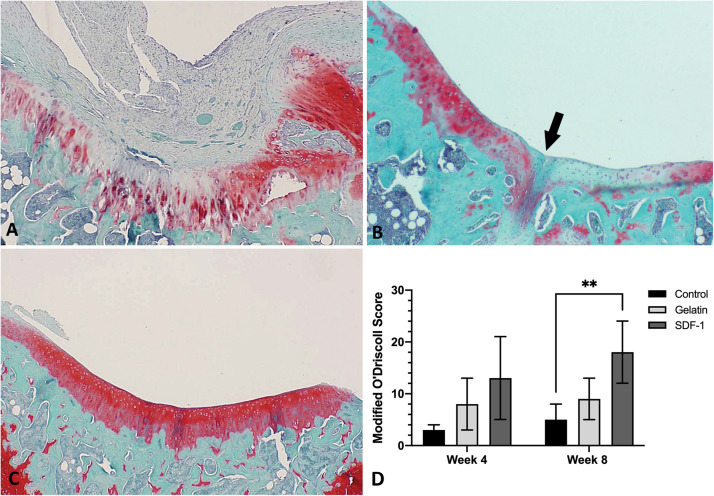
Histological evaluation with Safranin O/Fast Green to compare the repair of microfracture defects with implantation of gelatin–collagen I scaffolds carrying stromal cell-derived factor-1 (SDF-1) (50 ng/ml) and control groups *in vivo* (×40 magnification) at 8 weeks post-operatively. Representative images were selected based upon the best quality of cartilage repair within each group. **(A)** Defect only group. **(B)** Gelatin only group. **(C)** Gelatin and SDF-1 group. **(D)** Quantitative comparison of cartilage repair quality with Modified O’Driscoll scores at 4 and 8 weeks post-operatively. ^∗∗^*P* < 0.01.

No post-operative complications were noted throughout the study period. SDF-1-treated defects showed complete filling of mature cartilage tissue with strong proteoglycan content, smooth borders, and substantial incorporation into surrounding cartilage (Figures 3A–C). Modified O’Driscoll scores at 4 weeks post-operatively showed a trend toward improvement in cartilage repair quality in the SDF-1 group relative to the empty control group (13 ± 8 *vs*. 3 ± 1; *P* = 0.07); however, the difference was not statistically significant. No significant difference in cartilage repair quality was found at 4 weeks post-operatively between the SDF-1 group and the gelatin-only group (8 ± 5; *P* = 0.26). Modified O’Driscoll scores at 8 weeks post-operatively showed a significant improvement in cartilage quality in the SDF-1 group relative to the empty control group (18 ± 6 *vs*. 5 ± 3; *P* = 0.009) and a trend toward improvement when compared with the gelatin-only-scaffold group (9 ± 4; *P* = 0.09); however, the difference was not statistically significant. No significant difference in O’Driscoll scores was found when comparing the empty control and gelatin-only-scaffold groups at 4 weeks (*P* = 0.14) or 8 weeks post-operatively (*P* = 0.17; Figure 3D).

## Discussion

Microfracture is one of the most widely used techniques for the repair of articular cartilage (Oussedik et al., 2015; Wylie et al., 2015). However, microfracture results in filling of the cartilage defect with fibrocartilage, which is less durable than native hyaline cartilage and prone to re-injury (Krych et al., 2012; Xing et al., 2013; Oussedik et al., 2015). Implantable cell-free scaffolds coated in chemoattractive factors may improve the quality of cartilage repair following microfracture (Mishima and Lotz, 2008; Chanda et al., 2010; Fong et al., 2011; Eseonu and De Bari, 2015; Truong et al., 2017; Lu et al., 2018). In this study, we determined the optimal concentration of SDF-1 for MSC chemoattraction *in vitro* and found that incorporating SDF-1 into an implantable scaffold yielded a superior quality of cartilage repair when compared with microfracture alone.

SDF-1 promotes MSC migration and homing to the bone marrow through its receptor, CXCR4 (Shi et al., 2007; Baek et al., 2011). In addition to MSCs, CXCR4 is also expressed on hematopoietic progenitor cells, chondrocytes, and fibroblasts (Kucia et al., 2005; Villalvilla et al., 2014). As the SDF-1/CXCR4 axis is not exclusive to MSC homing, determining the optimal dosage of SDF-1 for the homing of MSCs to cartilage defects is necessary. Previously, a concentration of 50–200 ng/ml was reported to enhance the chemoattraction of rat BMSCs *in vitro*. However, when the concentration of SDF-1 was increased to 400 ng/ml and above, the chemoattractant effect began to significantly decrease (Ji et al., 2013). In our study, we confirmed the optimal concentration of SDF-1 to be between 10 and 100 ng/ml.

To apply SDF-1 *in vivo*, we used a gelatin carrier system developed for the application of growth factors (Dines et al., 2007, 2011; Uggen et al., 2010; Cummings et al., 2012). *In vitro* testing revealed that the release of growth factors from the carrier system could be sustained at a standard rate within the first 48 h (Uggen et al., 2010; Cummings et al., 2012). After implantation, the growth factors carried by the system maintained their functional effect *in vivo* (Dines et al., 2007; Uggen et al., 2010; Cummings et al., 2012). In our study, the gelatin–SDF-1 solution was loaded on a bovine collagen I scaffold for implantation in a rat microfracture model. When assessing the compatibility of MSCs with collagen scaffolds, type I collagen scaffolds exhibited superior attachment of BMSCs and cartilage-derived MSCs than type II collagen scaffolds. Moreover, the cells attached on the type I collagen scaffold maintained their spindle shape as a monolayer, while the cells on the type II collagen scaffold were round and tightly packed (Zhang et al., 2013). In clinical application, type I collagen scaffolds combined with hydroxyapatite showed improvement in the repair of cartilage defects (Filardo et al., 2013; Delcogliano et al., 2014).

In our study, implantation of an SDF-1-coated scaffold at the site of microfracture resulted in a significant improvement in cartilage repair, with results seen as early as 4 weeks post-operatively. Similarly, using a rabbit model, Chen et al. (2015) found that type I collagen scaffolds carrying SDF-1 (100 ng/ml) enhanced the repair of cartilage defects after 12 weeks. Zhang et al. (2013) utilized collagen I scaffolds carrying SDF-1 (120 ng/ml) on a partial-thickness cartilage defect using a rabbit model and found improved repair quality in the SDF-1 treatment group at 6 weeks post-operatively. In comparison to previous studies, our SDF-1 carrier system achieved nearly complete cartilage defect filling at 8 weeks post-operatively using an SDF-1 concentration of 50 ng/ml. A subsequent elution profile of our scaffold revealed an initial bolus release of SDF-1, followed by a continuous, steady release over the following 5 days, consistent with available literature (Sun et al., 2016). This may explain why we were able to achieve significant results at a lower concentration than those of other studies. While low SDF-1 levels are tied to healing, high SDF-1 levels have been paradoxically implicated in rheumatoid arthritis and osteoarthritis (Villalvilla et al., 2014; Kanbe et al., 2015). Thus, maintaining therapeutic levels of SDF-1 in the joint space by optimizing the biomechanics of the delivery system is vital.

The limitations of our study include the small sample size and relatively short post-operative time points. We found no significant difference in the quality of cartilage repair between groups at 4 weeks post-operatively as measured by Modified O’Driscoll scores. However, significant improvement in repair was noted at 8 weeks post-operatively in the SDF-1-treated group when compared with the empty defect group. When compared with the gelatin-dipped scaffold group, the SDF-1-treated group showed a tendency for improvement in the Modified O’Driscoll scores, but the difference did not meet statistical significance. In future studies, conducting analyses at longer time points, such as at 12- or 16-weeks post-operatively, may allow for a more appropriate length of time for mature tissue healing. Furthermore, increasing the sample size of the study would generate greater power to elucidate the difference between the gelatin- and SDF-1-treated scaffold groups. Immunohistochemical staining of healed tissue for collagen types I and II would provide definitive evidence of repair with hyaline *vs.* fibrocartilage; fibrocartilage contains a significant amount of type I collagen, while hyaline cartilage contains little to none. Based on our *in vitro* cell migration studies, a concentration of 50 ng/ml SDF-1 was chosen for the *in vivo* studies to optimize chemotaxis to the defect site with a minimal dose. However, we recognize that the kinetics of SDF-1 *in vivo* may differ significantly from the kinetics observed in *in vitro* studies. Future areas of study may include comparing a variety of SDF-1 concentrations *in vivo* to confirm the ideal concentration for cartilage repair and performing tests of durability and mechanical loading to provide evidence of clinically meaningful results. Other factors commonly implicated in cartilage repair, such as granulocyte-macrophage colony-stimulating factor transforming growth factor beta, platelet-rich plasma, and fibroblast growth factor, should be compared directly to and in combination with SDF-1 to formulate an optimal cocktail for cartilage repair (Kang et al., 2008; Huh et al., 2014; Harada et al., 2015; Truong et al., 2017).

In this study, we successfully demonstrated a cost-effective, one-step process for enhancing microfracture to improve the outcome of cartilage repair. We confirmed the chemotactic properties of SDF-1 on rat MSCs *in vitro* and found an optimized dosage range for chemotaxis between 10 and 100 ng/ml, consistent with previous literature. Furthermore, we successfully demonstrated a simple method of incorporating SDF-1 into a biocompatible scaffold at the site of osteochondral defect. SDF-1-treated defects displayed robust resurfacing with hyaline-like cartilage with minimal fibrous tissue compared with the control groups. Overall, the results suggest that scaffolds treated with SDF-1 can improve the quality of cartilage repair in an osteochondral defect.

## Data Availability Statement

The raw data supporting the conclusions of this article will be made available by the authors, without undue reservation.

## Ethics Statement

The animal study was reviewed and approved by the Institutional Animal Care and Use Committee of the Feinstein Institutes for Medical Research.

## Author Contributions

HL, NS, and DG contributed to the concept, design, and supervision of the study. TM, JS, PP, and HL collected, analyzed, and interpreted the data. TM, HL, and DG were involved in the writing and critical review of the manuscript. TM and DG conducted the final editing and proofreading. All the authors read and approved the final manuscript.

## Conflict of Interest

The authors declare that the research was conducted in the absence of any commercial or financial relationships that could be construed as a potential conflict of interest.
